# Molecular identification and geographic origin of a post-Medieval elephant finding from southwestern Portugal using high-throughput sequencing

**DOI:** 10.1038/s41598-020-75323-y

**Published:** 2020-11-06

**Authors:** Nikolaos Psonis, Carlos Neto de Carvalho, Silvério Figueiredo, Eugenia Tabakaki, Despoina Vassou, Nikos Poulakakis, Dimitris Kafetzopoulos

**Affiliations:** 1 Foundation for Research and Technology-Hellas (FORTH), Institute of Molecular Biology and Biotechnology, Ancient DNA Lab, N. Plastira 100, Vassilika Vouton, 70013 Irakleio, Greece; 2 Naturtejo UNESCO Global Geopark-Geology Office of the Municipality of Idanha-a-Nova, Centro Cultural Raiano, Av. Joaquim Morão, 6060-101 Idanha-a-Nova, Portugal; 3 Instituto D. Luiz-IDL Ciências da Terra, Faculty of Sciences of the University of Lisbon, Campo Grande, 1749-016 Lisboa, Portugal; 4 Polytechnic Institute of Tomar, Quinta do Contador, Estrada da Serra, 2300-313 Tomar, Portugal; 5 Portuguese Center of Geo-History and Prehistory, Largo de São Caetano, 2150-265 Golegã, Portugal; 6 Geosciences Center-University of Coimbra, Rua Sílvio Lima, University of Coimbra, 3030-790 Coimbra, Portugal; 7 Natural History Museum of Crete, School of Sciences and Engineering, University of Crete, Knosos Avenue, 71409 Irakleio, Greece; 8 Department of Biology, School of Sciences and Engineering, University of Crete, Vassilika Vouton, 70013 Irakleio, Greece

**Keywords:** DNA, Genomic analysis, High-throughput screening, Sequencing, Genomics, Sequencing, Genome informatics, High-throughput screening, Phylogeny, Archaeology, Evolutionary genetics, Palaeontology, Phylogenetics, Taxonomy, Evolutionary biology, Genomics, Haplotypes, Population genetics, Sequencing

## Abstract

Molecular species identification plays a crucial role in archaeology and palaeontology, especially when diagnostic morphological characters are unavailable. Molecular markers have been used in forensic science to trace the geographic origin of wildlife products, such as ivory. So far, only a few studies have applied genetic methods to both identify the species and circumscribe the provenance of historic wildlife trade material. Here, by combining ancient DNA methods and genome skimming on a historical elephantid tooth found in southwestern Portugal, we aimed to identify its species, infer its placement in the elephantid phylogenetic tree, and triangulate its geographic origin. According to our results the specimen dates back to the eighteenth century CE and belongs to a female African forest elephant (non-hybrid *Loxodonta cyclotis* individual) geographically originated from west—west-central Africa, from areas where one of the four major mitochondrial clades of *L. cyclotis* is distributed. Historical evidence supports our inference, pointing out that the tooth should be considered as post-Medieval raw ivory trade material between West Africa and Portugal. Our study provides a comprehensive approach to study historical products and artefacts using archaeogenetics and contributes towards enlightening cultural and biological historical aspects of ivory trade in western Europe.

## Introduction

Identification of unknown individuals at the species level is of great interest in many scientific areas^1^ and references therein. Especially in archaeology and palaeontology its usefulness is evident in cases where the taxonomic determination is obscured due to lack of morphological diagnostic characteristics e.g. Ref.^2^ and to high morphological variation among and within species e.g. sexual dimorphism; see Ref.^3^. Even more helpful is the application of genetic data on determining the geographic origin of an animal remain or product of trade. The utility of molecular methods to establish the provenance of wildlife products has been demonstrated through their application to elephant ivory poaching and seizuring following an approach of direct amplification of specific genes (especially mitochondrial) or microsatellites e.g. Refs.^4,5^.

The development of high-throughput sequencing (HTS) techniques has revolutionized the way we explore biological diversity (for a review of methods see Ref.^6^). Genome-skimming, also known as low-coverage whole-genome shotgun sequencing is a cost-effective technique, capable to recover high-copy parts of total genomic DNA, such as organellar genomes, at low sequence depth^7,8^. This method has been proven efficient for species identification and phylogenetic studies on centuries-old museum samples^9^ and references therein, as well as on raw walrus ivory material^10^. This type of biological material display characteristics of ancient DNA (i.e. fragmented DNA molecules, altered sequence information due to cytosine deamination) that are not well-handled by conventional protocols, such as PCR (i.e. due to high rates of unsuccessful amplification and/or contamination), rendering genome skimming a valuable alternative e.g. Refs.^11,12^. Under this scheme, genome skimming also enables the post-mortem DNA damage profiling of the sample’s DNA and monitors for contamination, evaluating in this way the authenticity of degraded DNA and the levels of macromolecular preservation.

Here, we combine ancient DNA methods and genome skimming on a historic elephantid molar tooth found in the southwestern Portugal coast, in order to identify the species that belongs, and narrow down its geographic origin. To achieve this goal, we performed molecular species identification using a competitive mapping, as well as a metagenomics approach, whereas the sample’s providence was circumscribed through the phylogenetic placement of its mitochondrial DNA consensus sequence into the phylogenetic tree of extant elephants containing the majority of discrete clades and subclades discovered over the last two decades^13^. Taking into account these results, the present study attempts to enlighten some cultural and biological historical aspects of ivory in west Europe, relating to the trading networks, market and consuming of products and artefacts.

## Materials and methods

### Sample information and radiocarbon dating

The molar tooth was found close to the mouth of the Mira River, located at the SW coast of Portugal, Vila Nova de Milfontes town; geographical coordinates 37° 43′ 35.73″ N–8° 46′ 28.43″ O (see Supplementary Fig. S1). The tooth was brought to our knowledge as a random finding in the tidal flat made by a fisherman. In southwest coast of Portugal rare bones and tracks attributed to the straight-tusked elephant *Palaeoloxodon antiquus* have been found in Late Pleistocene aeolianites^14^, exposed in the coastal cliffs of Vila Nova de Milfontes^15^. During the 1980s, a few kilometres south of the mouth of Mira River a fishing trawler came up with elephant tusks identified as belonging to the forest savanna elephant *Loxodonta cyclotis*^16^, together with a fish amphora of African origin, suggesting the presence of a shipwreck 300 m deep, dated from the 2nd Iron Age (Cardoso, *op. cit.*). The new finding of an isolated molar tooth was obviously interesting because it could be related either to some of the last *Palaeoloxodon* elephants living in Europe during the Last Glacial period, preserved as a body fossil, or to ancient/historic trade of raw ivory.

Notwithstanding, samples of the tooth enamel were surprisingly dated of 230  ± 30 BP (Beta—548645, 2020) BetaCal3.21: HPD method: INTCAL13 + NHZ2. Calibrate date: (95.4%) 1955–1956 cal AD (6–7 cal BP). The radiocarbon date was made in Beta Analytic lab using 4 in-house NEC accelerator mass spectrometers and 4 Thermo IRMSs. "Conventional Radiocarbon Age" used by this lab for calendar calibration is the Libby half-life (5568 years), corrected for total isotopic fraction.

### Morphological identification

The tooth found shows some curious morphological features (see Supplementary Fig. S2). Details on the morphological characteristics can be found in the Supplementary Note S1. The cranial-dental morphological phylogenetical analysis of the Elephantidae^17^ has shown that the wide range of metric variation in individual teeth in the African elephant group, as well as the Eurasian species complicates comparisons between species^18^. *Loxodonta cyclotis* teeth are virtually indistinguishable from that of *L. africana*^19,20^. The retention of a “loxodont sene” in extinct elephants such as *P. antiquus*, *Elephas recki*, and *E. namadicus*, as well as the dwarfed Mediterranean species, suggests a close evolutionary relationship with *Loxodonta*, as do other cranial similarities^17^.

### Ancient DNA analysis and “shotgun” sequencing

The samples for DNA analysis were taken in LAP (the Archaeozoology and Palaeontology laboratory of Portuguese Center of Geo-History and Prehistory) using a sterilized HS saw blade in an electric mini drill. All ancient DNA sample processing, DNA extraction and libraries preparation were performed in cleanroom facilities, physically isolated from other laboratories and exclusively dedicated for ancient DNA analysis [Ancient DNA Lab of the Institute of Molecular Biology and Biotechnology (IMBB), Foundation for Research and Technology-Hellas (FORTH), Irakleio, Crete, Greece] following strict ancient DNA guidelines e.g. Ref.^21^, whereas the libraries amplification and Next Generation Sequencing was performed in the Genomics Facility of IMBB-FORTH.

A total of three different pieces from the molar was processed. Two of them were found loosen inside the dental root cavities (aDNA Lab codes: ADNA_100098_1-2) and the third was a fresh cut piece of a lamella root consisting of both tooth cementum and dentin (ADNA_100098_3). Sample processing, DNA extraction, and blunt-end library preparation were performed following established procedures^22^ with a few modifications as described in the Supplementary Note S2. The indexed libraries were ‘shotgun’ sequenced on an Illumina NextSeq 500 platform using 75 + 6 bp single read chemistry.

### Ancient DNA data assessment

The raw DNA data handling included de-multiplexing, quality control estimations, and adapter removal. The presence of genomic elephant DNA was achieved by mapping the reads against the reference genome of the African Savanna elephant. The mapped reads were filtered, duplicates were removed, and the indels were re-aligned. Details on the above procedures can be found in the Supplementary Note S3. Read depth and coverage were determined using Qualimap 2 v.2.2.1^23^. To investigate the level of DNA degradation in our samples, the mapped sequences were analyzed with mapDamage2.0 v.2.0.8^24^. The above analyses were performed using the raw data from each sequenced library separately (for comparisons among the dental tissue extracts), as well as from the merged raw data. Only the merged data were used for the rest of the analyses.

### Genetic sex estimation

The determination of the genetic sex of the specimen was performed by utilizing the ratio of the mapped reads in sex and autosomal chromosomes. More precisely, given that the reference genome used does not include the Y chromosome (female), we calculated the normalized ratio of the mapped reads in the X chromosome (length = 120,050,768 bp) to those mapped in chromosome 8 (length = 128,409,435 bp) that has almost the same size as the X chromosome. We achieved normalization by dividing the number of mapped reads by the chromosome length. If the normalized ratio was close to 1.0 then the sample was assigned as female since the female has two X chromosomes, as well as two chromosomes 8 given that the elephants are diploid organisms. If the ratio is closer to 0.5 then the sample was assigned to male (one X chromosome and two chromosomes 8).

### Molecular species identification

FastQ Screen v.0.13.0^25^ allows for the same sequencing library to be easily aligned to multiple reference genomes using Bowtie v.1.2.2^26^. The percentage of raw reads that aligned (a) uniquely to a single genome, (b) to multiple places in the same genome, (c) uniquely in multiple genomes, and (d) to multiple places in multiple genomes can then be assessed. This method has been successfully used for the molecular species identification of ancient specimens, including medieval parchments made of livestock-skin^27^. Since reference genome is currently available for only one elephant species, we used the reference mitochondrial genomes instead, which is available for seven extinct and extant elephants. The mitochondrial DNA species assignment was also assessed via a metagenomics approach using MALT v.0.4.0^28^ and the entire mitochondrial DNA database (mito_nt) of GenBank (https://www.ncbi.nlm.nih.gov/genbank/). Details on both the above procedures can be found in the Supplementary Note S4.

Given that the two African elephant species are known to hybridize^29^ and references therein, the mtDNA is not an adequate molecular marker to distinguish these two species as it is inherited only by females to their sprouts. In an attempt to distinguish between the two African elephant species, we repeated the FastQ Screen analysis using low coverage genomes of *L. cyclotis* and *L. loxodonta* (details in Supplementary Note S4).

Finally, to test whether the tooth belongs to a pure or a hybrid African elephant, we used the F3 statistics, which test if a target population (C; the tooth finding) is admixed between two source populations (A and B; the two African elephant species). Details on F3 Statistics are given in Supplementary Note S5.

### Mapping to reference mitogenome and mtDNA consensus sequence generation

After the identification of the focal specimen in species level according to mtDNA, the raw trimmed and length filtered reads were also aligned to the corresponding reference mitogenome following the same procedure as in “Ancient DNA data assessment”. Two consensus mitochondrial DNA sequences were produced using ANGSD v.0.925-15-g334e8da^30^ with and without employing a minimum site depth filtering (details can be found in the Supplementary Note S6). Downstream phylogenetic analyses used both the manually corrected mtDNA consensus sequence and the one filtered with a site depth of 3.

### Alignment and mitochondrial DNA phylogenetic tree reconstruction

For the mtDNA phylogenetic reconstruction we added the two consensus mtDNA sequences (see “Molecular species identification” above) into two datasets formed using previously published sequences (see Supplementary Table S1). The first one, consisting of 33 individuals, included all the complete mitogenome sequences of the GenBank that was assigned to a Proboscidean, but including only three representative *Mammuthus* samples (one *M. columbi* and two *M. primigenius*). This dataset was used to place with high confidence our specimen to one of the main phylogenetic clades/subclades. The second dataset, consisting of 654 individuals, comprised all the African (and one Asian) elephant sequences in Genbank covering a continuous 4258 bp mtDNA fragment (positions 11,750–16,006 in the African savanna elephant reference mtDNA genome). This dataset was used to narrow down the provenance of our focal specimen, as it covers a great proportion of the elephant distribution and mtDNA diversity in the entire Africa, including 13 countries^13^. After aligning the sequences, we performed estimation of partition and nucleotide substitution model and we reconstructed Neighbor Joining and Maximum Likelihood phylogenetic trees (details in Supplementary Note S7).

## Results

### Sequencing statistics, mapping to reference genome and DNA preservation

Statistics of the ‘shotgun’ sequencing are presented in Supplementary Table S2. Following bioinformatic trimming and mapping against the *L. africana* reference genome between 23.64% and 63.48% of the retained sequences could be aligned uniquely (endogenous content). These values indicate a very good DNA preservation on all three samples. When analyzed in mapDamage2, the uniquely mapped, non-clonal sequence data from all DNA extracts showed a marked increase (7.69–11.00%) in C to T deamination damage towards the 5′-ends of the sequences, as well as short average length (57.81–60.31 bp), typical for degraded DNA e.g. Ref.^31^ (Supplementary Table S3 and Supplementary Fig. S3).

### Genetic sex and molecular species identification

The normalized ratio of X to 8 chromosome mapped reads, which was used to estimate the genetic sex was 0.92, as 51,140 and 59,480 non-clonal sequence reads were uniquely mapped to chromosome X and 8, respectively. This indicates that the tooth sample belongs to a female elephant.

**Figure 1:**
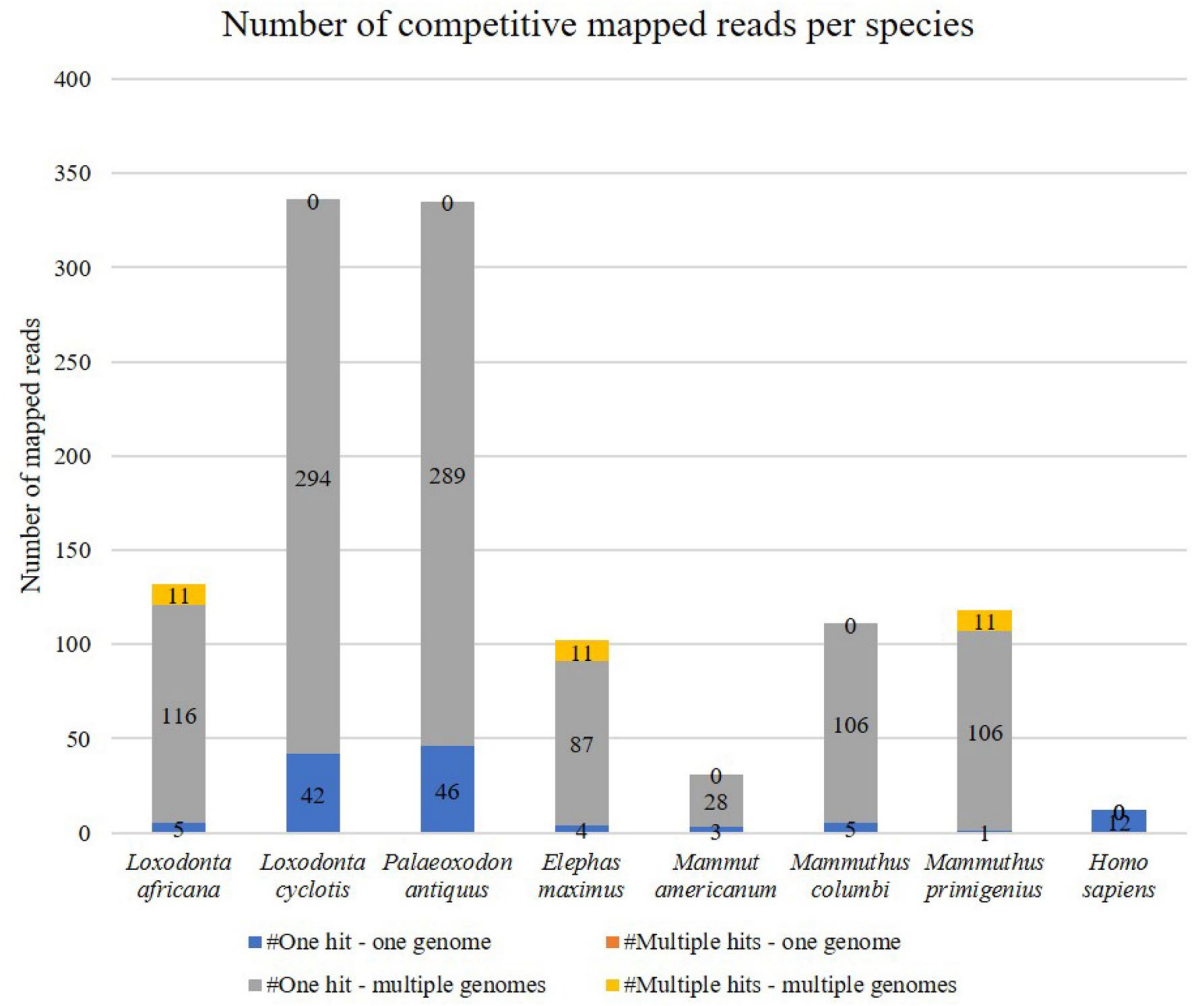
Competitive mapping (FastQ Screen analysis) of the generated sequences from the elephant tooth against the reference mitochondrial genomes of all three living elephant species and four extinct ones, as well as against the reference mitogenome of humans. No “multiple hits – one genome” were observed in all cases (zero values not shown).

According to both the FastQ Screen and MALT analyses (see Fig. 1 and Supplementary Fig. S4) the mitochondrial DNA of the tooth is more similar either to the African forest elephant (*L. cyclotis*) or to the straight-tusked elephant (*P. antiquus*). Given that the tooth dates back to ca. 300 y. o. and that *P. antiquus* became extinct during the Upper Pleistocene from Portugal^14,32^, the species assignment based on the mtDNA is narrowed down to *L. cyclotis*.

When using the entire genomes of the two African elephant species in a FastQ Screen analysis, in order to investigate if the tooth belongs to a “pure” forest elephant or to a hybrid, the results showed a much higher percentage of total reads being mapped to *L. cyclotis* than to *L. africana*, whereas the same was observed when accounting the uniquely mapped reads to one genome only (see Supplementary Fig. S5). Moreover, according to the F3 statistic (F3 = 0.133746; Z-score = 5.25377), no evidence was found to suggest that the tooth belongs to an individual that was the product of admixture between the two African elephant species. Thus, according to this analysis we infer that the tooth specimen found belongs to a pure African forest elephant individual.

### Mapping to the reference mitogenome, alignment and phylogenetic reconstruction

In total, genome skimming achieved a 3.19X mean depth of mitogenome coverage. Out of the 16,109 bp of the reference mitogenome, 6.83% and 45.01% of the sites had 0 and less than 3 depth of coverage, respectively. The manually corrected consensus sequence (see “Mapping to reference mitogenome and mtDNA consensus sequence generation”) had 7.10% zero covered or masked sites.

Descriptive statistics for both the alignments can be found in Supplementary Table S4.

The manually corrected consensus sequence had 6.74% and 8.52% missing data, for the full and the partial mitochondrial dataset, respectively, whereas the site depth = 3 filtered consensus sequence had 44.81% and 48.47% missing data, respectively. The best-fit partitioning scheme for the ML analyses resulted in 7 subsets (lnL =  − 39,208.84; BIC value: 79,395.55) and 4 subsets (lnL = − 9741.92, BIC value: 30,631.48) for the two datasets, respectively. Their selected nucleotide substitution models are summarized in the Supplementary Tables S5 and S6, respectively.

**Figure 2:**
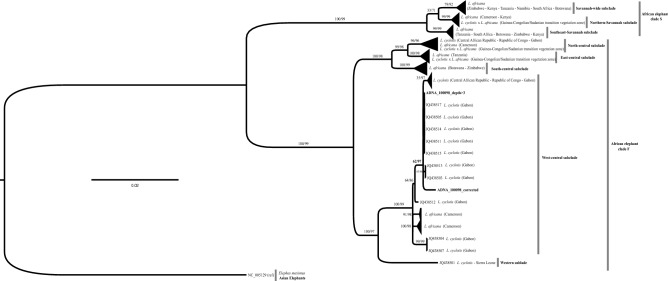
The placement of the tooth (ADNA_100098) mtDNA consensus sequences in the Maximum Likelihood phylogenetic tree of *Loxodonta* using the partial mtDNA genome dataset continuous 4,258 bp fragment). The numbers on the branches correspond to the bootstrap support (NJ/ML). The clade/subclade nomenclature and the comment inside brackets is based on Ref^13^.

Both phylogenetic analyses (NJ, ML) on both the full and the partial mtDNA datasets produced trees with very similar topologies (see Supplementary Fig. S6 and Fig. 2, respectively). Maximum likelihood analysis resulted in a topology with lnL = − 40,010.50 with bootstrapping convergence after 50 replicates for full mtDNA dataset, whereas the corresponding values for the partial mtDNA dataset were lnL = − 9835.56 and 200 bootstrap replicates. According to resulted tree for the full mtDNA dataset the tooth sample belongs to the F subclade of the African elephant, and more precisely to the west-central subclade. The partial mtDNA based resulted tree also supports the placement within the west-central subclade (F clade) and moreover indicates that the specimen is more closely related to individuals from Gabon, the southwestern part of Central African Republic, and the Republic of Congo (Congo-Brazzaville). The two consensus sequences did not differ concerning their phylogenetic placement.

## Discussion

### Molecular species identification

The family Elephantidae includes three extant elephant species, the Asian elephant (*E. maximus*) distributed in Asia^33^, the African savannah elephant (*L. africana*) with a patchy distribution in Sub-Saharan Africa (mainly East, Central and Southern Africa), and the African forest elephant (*L. cyclotis*) occurring only in Central/Central-west Africa^34^. Considering the fossil record, many proboscideans are recognized, dating from the Late Paleocene. Ancient DNA (draft genomes) is known from four Pleistocene extinct species, the American mastodon (*Mammut americanum*), the wooly mammoth (*Mammuthus primigenius*), the Columbian mammoth (*M. columbi*), and the straight-tusked elephant (*P. antiquus*)^35^ and references therein.

One of the main scopes of the present study was to unambiguously assign the tooth to a species. According to the mtDNA-based FastQ Screen and MALT analyses and given the ^14^C dating, the post-medieval tooth belongs to *L. cyclotis*, despite the fact that many reads were aligned also to the extinct *P. antiquus*. This finding is not surprising, as a recent paleogenomic mtDNA-based phylogenetic study^18^ shown that *P. antiquus* has high genetic affinity with *L. cyclotis* and more precisely that it is phylogenetically placed within the clade that includes *L. cyclotis*, a non-monophyletic relationship inferred here, too (see Supplementary Fig. S6). However, this relationship is confounded into the mitochondrial tree, as the whole-genome species tree supports two closely-related, but monophyletic species^35^. The mitonuclear phylogenetic incongruence is attributed to ancient (~ 3–0.1 mya) hybridization between straight-tusked and African forest elephants that possibly occurred in Africa^35^.

A major difficulty to determine the species status of the molar tooth was to distinguish if the tooth belongs either to an anatomically African forest elephant, to a hybrid, or to a savanna elephant with a remnant *L. cyclotis* mitogenome (conplastic individual) as a result of recurrent backcrossing causing mitonuclear dissociation. This issue is originated from the fact that despite the two African elephant species are genetically distinct^35,36^, they share hybridization zones^29,37^. The two species occupy different habitats with little overlap in the transition forest-savanna zones, where both elephants may be present and where it has been found that they hybridize producing fertile offspring^29,37^. As a result, F1 hybrid individuals carry the matrilineally inherited mitochondrial DNA of one species and the patrilineally inherited Y-chromosomal DNA of the other, whereas their autosomal and X-chromosomal DNA will belong to both species. Recurrent backcrossing between hybrids and pure species may also result to conplastic individuals that carry the mitochondrial DNA of one species and the nuclear DNA (X-, Y-chromosomal and autosomal DNA) of the other (evident in the partial-mtDNA tree of Fig. 2). Traditionally, the discrimination among the above classes is achieved by studying different molecular markers located on each of the above uni- and bi-parentally inherited genomic parts e.g. Refs.^29,37^. However, none of the nuclear or Y-linked DNA markers used in previous studies where covered by our genome skimming method applied in the tooth finding. As an alternative, we perform a FastQ Screen and a F3 statistics analysis using two low-coverage African elephant genomes. The FastQ Screen analysis rejected the conplastic individual hypothesis (savannah elephant with forest mitogenome) as the majority of the one-genome mapped reads were aligned to *L. cyclotis*. The presence of a substantial number of reads mapped only to *L. africana* could indicate either that the sample belong to a hybrid or it is an artifact of the analysis as the low-coverage genomes used are not high-quality assemblies. As a result, some of the reads mapped to *L. africana* genome could have been mapped to genomic areas that are not well represented or totally absent in the *L. cyclotis* genome. Nonetheless, the F3 statistics did not supported the hybrid individual hypothesis, *ergo* we infer that the tooth belongs to a pure African forest elephant. Our inference is also supported by the fact that a limited number of hybrid individuals have been observed^29,37^, whereas the hybridization zones are considered to be a response to human disturbance^29^, which was intensified in sub-Saharan Africa during the last 200 years e.g. Refs.^38,39^, a century after our focal individual was alive, according to the ^14^C dating. There is no evidence of ancient hybridization between the two African elephant species as inferred by whole-genome comparison^35^.

### Genetic evidence of specimen geographic origin

Given that the single tooth was found in the southwestern Portugal coast and it is dated at post-medieval times, one major question concerns the provenance of the finding. Mitochondrial DNA markers has been found to constitute a useful tool for the triangulation of African elephant provenance, in combination with the use of nuclear markers, such as microsatellites^13^. The effectiveness of mtDNA comes from an intrinsic characteristic of elephant behavior, known as matrilocality or female philopatry, according to which the female elephants, in contrary to the males, do not migrate between elephant herds, but rather stay with the natal herd e.g. Ref.^40^. As a result, the mtDNA phylogeography differs from the nuclear one, as the mitochondrial genome is tied to the geographic range of the herd, whereas nDNA phylogeography is subject to the male-mediated gene flow among herds^13,41^. The fact that our specimen was identified as female, strengthens its provenance estimation.

The mtDNA-based species identification performed in the present study was able to exclude Asia as a broad geographic origin. Moreover, by inferring that the focal individual was a pure *L. cyclotis* we narrowed down the geographical origin of the tooth from the entire sub-Saharan Africa to the central-west part, where forest elephants are currently and were historically distributed (based on the distribution of tropical forests). Moreover, according to the full mitochondrial phylogenetic tree (see Supplementary Fig. S6) the tooth’s mitogenome is grouped in the F clade of African elephants. The F clade includes five major subclades, with *L. cyclotis* individuals being present in four of them, namely the western, the west-central, the north-central, and the east-central^13^. Both the full- and the partial-mtDNA tree (see Supplementary Fig. S6 and Fig. 2) show that phylogenetically the molar tooth falls in the west-central subgroup that includes African forest individuals from Gabon, the southwestern part of Central African Republic, and the Republic of Congo (Congo-Brazzaville). According to Ishida, et al.^13^ this subclade is also shared among forest elephants from Ghana and Ivory Coast. Based on this, far western countries such as Guinea, Sierra Leone, and Liberia (western subclade) are excluded, whereas the same applies for far northern, far eastern, and far southern central-west countries like the Democratic Republic of Congo (Congo-Kinshasa) and Uganda (north-central and east-central subclades). Thus, the provenance of the tooth could be delimited in the aforementioned five countries, albeit a few more countries should be included in the list as potential geographic origins, due to lack of reference genetic evidence or due to the difference between the current and the historical distribution^42^ of forest elephants. Thus, countries in which the west-central clade is not currently present (Cameroon), or in which the forest elephant populations have been vastly declined during the last century (Equatorial Guinea, Nigeria, Benin, and Togo), but are located between the countries that the central-west subclade is distributed, should be also considered as potential sources.

### Specimen dispersal

Regarding the question on how this tooth have ended up in the coast of southwestern Portugal (close to the mouth of Mira river), only speculations can be made. However, it is interesting to discuss the potential ways of dispersal and order them by their likelihood, given the ^14^C dating of the specimen (~ 300 y. o.) and the genetic and other pieces of evidence provided here. Four main hypotheses could be made (along with their combinations): (a) active dispersal of the animal (b) translocation of the alive individual by humans, (c) passive dispersal of remains by the sea currents, and (d) human translocation of remains, including ivory trade import.

The active dispersal of elephants from Africa to Portugal, which are known to be excellent long-distance (> 40 km) swimmers e.g. Ref.^43^, is not supported, as North-African elephants (*L. a. pharaohensis*) that resembled *L. cyclotis* became extinct in Roman times^44^. Moreover, the sea current system in Portugal (Portugal Current) is flowing southeastern, from the Iberian shores to North-West Africa^45^, which means the passive dispersal of a dead animal’s remains by sea currents from West Africa to Portugal is also unlikely. Based on these and on the finding of a single molar tooth, the hypotheses of human translocation and human ivory trade import seem more likely. The ivory trading in Iberian Peninsula started as early as the Chalcolithic times e.g. Ref.^46^. Schuhmacher and Banerjee^46^ identified eight objects from archeological sites of this period in Portugal, all of them attributed to *L. africana* except one case related to the use of much older *P. antiquus* ivory. The ivory trade in the Iberian Peninsula would continue during the Phoenician and Roman times with solid evidences, and later with the arrival of the Portuguese to the coasts of Africa and India, after the fifteenth century CE. The scenario of importing the tooth through ivory trade in the eighteenth century shows some evidence, as the presence of alive elephants during the post-medieval times in Europe was limited, where they were used as luxury pet presents or exotic attractions in tours^44^.

The geographical origin of the sample from west/central-west Africa is in concordance with the establishment of sub-Saharan African ivory trade (among other types of trade, such as gold and slaves) during the Early Modern World, which started with the Portuguese exploration of west Africa coasts during the Age of Discovery (fifteenth century CE) and the subsequent creation of the Portuguese Colonial Empire^47^. In the fifteenth century CE, Portuguese explorers arrived on the West African coast and the Sapi people of Sierra Leone provided them the first pieces of ivory^48^. By the late seventeeth and early eighteenth centuries CE, Europeans were supplied with ivory from the entire West African coast, from Senegal to Cameroon and further south as far as Angola^39^. During the first quarter of the eighteenth century CE ivory was a luxury and the European demand increased during this period. This demand was met by increasing imports from West Africa as a result of the exertions of African hunters and traders^49^. From the eighteenth century CE, the sub-Saharan Africa became the main ivory source for Europe^50^. The fact that just a single tooth was found probably supports further the scenario of importing through ivory trade.

Finally, a scenario that supports the human translocation of either an entire living animal or ivory products is the dispersal of teeth after a shipwreck. Shipwrecks of the seventeenth century CE containing ivory have been reported in Namibia^51^ and India coasts^52^. The western coast of Portugal shows a large number of shipwrecks related to the Portuguese trading routes after the fifteenth century CE. Also, many European countries that followed Portugal in the global trading and territorial expansion in later centuries used the western Portuguese ports in their maritime routes. Off the coast near the mouth of the Mira River several shipwrecks are known since pre-Roman times. We looked for information about shipwrecks in the area that happened within the radiocarbon age range determined for the tooth and we came up with a single match. In the inventory of the authority responsible for the historical and cultural heritage in Portugal, the Direcção-Geral do Património Cultural, there is a reference to the disaster of the 90 tons French boat named “Diligent Postillon” in 1735, close to Vila Nova de Milfontes (https://arqueologia.patrimoniocultural.pt/index.php?sid=sitios&subsid=2687408). Almost no information was reported about the saved cargo and the exact location of the shipwreck is unknown. However, this boat belonged to Jacques Le Roy, a French captain and later businessman operating from the port of Nantes^53^. Jacques Le Roy was owner of several slave ships that operated between Cabinda (Angola), then a territory of Portugal in the mouth of Congo River, and other Portuguese colonies like Prince Island, providing slaves to French possessions in the Caribbean^54^.

Dispersal of elephant remains or ivory products from a eighteenth century CE shipwreck to the Portuguese coast could be attributed to the tsunami of 1755 that occurred after the Great Lisbon earthquake of magnitude 8.5–9.0 R and rose the sea level dozens of meters inland, rushed up the rivers and caused severe damages to several Portuguese and Spanish coastal cities^55^. Storms affecting the shores during spring tides could also have been responsible in bringing the tooth up the Mira River, especially if the shipwreck happened very close to the river mouth, since the specimen shows no evidence of abrasion related with long-term permanence on the sea bottom. Moreover, the analysis of the malacological content found in sediments trapped in the molar root canals indicated the presence of typical mediolittoral and infralittoral species, such as *Gibbula umbilicalis* and *Bittium reticulatum*, respectively.

### Conclusions and future perspectives

In the present study, we performed archaeogenetic genome skimming analysis in a molar tooth found in the southwestern Portuguese coast in order to identify the species and determine its geographic origin. As a conclusion to our scientific questions, the analyses performed indicate that the specimen found dates back to the eighteenth century CE, belongs to a female African forest elephant (non-hybrid *L. cyclotis* individual), and could be geographically originated from one of the following west and west-central African countries: most probably from Gabon, the southwestern part of Central African Republic, the Republic of Congo (Congo-Brazzaville), Ghana, the Ivory Coast, and potentially from Cameroon, Equatorial Guinea, Nigeria, Benin, Togo. Noteworthy, historical evidence link the tooth to eighteenth century CE Congo-Brazzaville.

Ivory has been considered as a desirable substance for millennia, not only for its beauty, but for its durability, malleability, and absence of splintering^50^. This specimen of raw ivory is an excellent example in which exotic animals, such as elephants, played a major role in many aspects of the European culture of the early eighteenth century as providers of raw material and as socio-economic and symbolic resources. Thus, our findings provide insights to a dynamic interaction between the cultures through commercial and trading activities.

Existing approaches to determine the species and/or geographic origin of elephant products (mostly tusk ivory) have been developed and successfully applied e.g. Refs.^13,56,57^. However, by using PCR amplified DNA markers these methods are not well-fitted for degraded samples (e.g. ancient or historic ivory artifacts or remains), as it has been demonstrated that the use of PCR with degraded DNA may produce false-negative (non-amplifiable DNA template), as well as false-positive results (contamination biases)^27^ and references therein. One of the main advantages of shotgun sequencing in archaeogenetics is that it allows to obtain ancient/historic DNA signature patterns^10,31^ and also to observe putative contaminated reads, as well as postmortem deamination-related misincorporated nucleotides and sequencing errors on sites covered by overlapping sequences, allowing to minimize their introduction into the final datasets. On the other hand, one of the main disadvantages of the method used in the present study is that it limits down the provenance of the sample only to regional areas or countries as it massively depends on the resolution power of mtDNA and the current mtDNA elephant phylogeography. One way to improve this weakness is to use hybridization capture enrichment techniques e.g. Ref.^58^ with sequence baits designed to capture both mtDNA and nDNA (autosomal and sex chromosomal) alongside other useful (i.e. phenotypic) markers capable of more precise triangulation of the geographic origin. Finally, further improvement of provenance precision on ancient or historic elephant samples, may be achieved by combining ancient DNA and non-DNA methods, such as isotopic analyses e.g. Ref.^59^.

## Supplementary information

Supplementary Information.

## Data Availability

The raw (fastq format) genetic data are available in the NCBI Short Read Archive (SRA) under the BioProject accession PRJNA662209 (SRA accessions SRR12606481 to SRR12606483). The manually corrected mtDNA consensus sequence is available in GenBank (Accession Number MW006629).
