# Supplementary material for: Molecular identification and geographic origin of a post-Medieval elephant finding from southwestern Portugal using high-throughput sequencing
Source: Sci Rep. 2020 Nov 6;10:19252. doi: 10.1038/s41598-020-75323-y (PMC7648095; doi:10.1038/s41598-020-75323-y)
Supplement: Supplementary file 1 — Supplementary Information. [file 41598_2020_75323_MOESM1_ESM.pdf]

SUPPLEMENTARY INFORMATION

Molecular identification and geographic origin of a post-Medieval elephant finding from southwestern Portugal using high-throughput sequencing.

Nikolaos Psonis, Carlos Neto de Carvalho, Silvério Figueiredo, Eugenia Tabakaki, Despoina Vassou, Nikos Poulakakis & Dimitris Kafetzopoulos

Supplementary Note S1. Details on the morphological characteristics of the molar tooth.

The tooth found shows some curious morphological features (**Fig. 2**). It is a right superior molar, according to the shape of the occlusal surface and way of curving of the tooth, although incomplete because it only shows seven lamella and is visibly damaged. It seems to be a M3 and displays a strong reduction of the width to the anterior edge and curve towards the posterior end (**Fig. 2**). The molar shows a high hypsodonty index of 2,14 (high and narrow tooth with greater height and width towards the posterior end), and the laminar frequency along the longitudinal plane of the molar is 5, with inclined plates from a side view, showing v-shaped valleys between them and a dot-dash-dot pattern of the asymmetrical enamel forming a pseudo-lozenge in the occlusal surface; enamel is <3 mm thick and enamel folding is regular and of low amplitude. The molar roots, despite broken, seem to open; the anterior root is followed by a space covered by one plate behind which the first set of “paired” roots lie.

Supplementary Note S2. Ancient DNA analysis and shotgun sequencing.

Sample processing and DNA extraction were performed following established procedures [1]. Briefly, in order to avoid contamination due to previous handling, the surface of the samples was mechanically removed using a diamond disk, and a small part was cut and crashed resulting into 60, 130, and 225 mg of powder for each piece, respectively. The powder samples were pre-digested in 2 ml of digestion buffer (0.47M EDTA, 10mM Tris-HCl pH = 8.0, 0.2 mg/ml Proteinase K (New England Biolabs, Ipswich, MA, USA) and 0.5% N-Laurylsarcosyl) for 15min to further remove contaminants [2] before full digestion with 3.5 ml digestion buffer for 24 hours at 42 °C. DNA extraction was performed using a silica suspension that was prepared as follows: 4.8 gr of silica (0.5-10 μm particle size) was mixed with 40 ml H_2O, followed by sedimentation for 1 hour. Thirty-nine ml were transferred to a new tube and were left to sentiment for 4 hours. The top 34 ml were carefully removed and silica was activated with 48 μl 37% HCl. The digested samples were mixed with 100 μL of silica and 40 ml of binding buffer (4.9 M Guanidinium Thiocyanate, 29.3% isopropanol, 24.9 mM NaCl and 87.6 mM sodium acetate, pH = 4) and pH was adjusted to 4-4.5 with 37% HCl and incubated at room temperature for 1 hour. After the end of the incubation, the supernatant was removed and the pelleted silica was resuspended in 1 ml of fresh binding buffer, spun

down, and washed twice with 1 ml 80% ice-cold ethanol. Finally, the DNA was eluted in 85 µl TEB buffer (10 mM Tris-HCl, pH 8.5 supplemented with 0.05% Tween-20). A negative extraction control (water blank) was included to monitor for contamination. The DNA concentration was measured in Qubit Fluorometer (Thermo Fisher Scientific, Waltham, MA, USA).

DNA extract was built into a blunt-end library according to procedures previously described [1] with a few modifications. The end-repair step was performed using the NEBNext End-repair module (E6050; New England Biolabs, Ipswich, MA, USA) with 21,75 µL of DNA extract, 2.5 µL end repair reaction buffer 10X, and 1.25 L end repair enzyme mix. The solution was incubated for 20 min at °12 C, followed by 15 min at 37 °C, and purified using 260 µl of the same binding buffer as used for extractions (see above) with Qiagen MinElute columns (Qiagen, Hilden, Germany), and eluted in 17 µL of EB buffer. Next, Illumina-specific adapters (Illumina, San Diego, CA, USA) were prepared as in Meyer and Kircher [3] and ligated using the NEBNext Quick Ligation module (E6056) to the end-repaired DNA in 25 µL reactions (15 µL of DNA, 5 µL of :Quick ligation buffer 5X, 0.5 µL of adapter mix, 2.5 uL of Quick T4 DNA ligase, 2 µL of H2O). This solution was incubated for 15 min at 20 °C and purified with 125 µl of PB buffer on Qiagen MinElute columns, before eluted in 25 µL EB buffer. The adapter fill-in reaction was performed in a 30 µL volume (23.5 µL of adapter ligated DNA, 3 µL of NEB ThermoPol® ReactionBuffer 10X, 2 µl of dNTPs 2.5 mM, and 1.5 µL of NEB Bst DNA polymerase, Large Fragment) and incubated for 20 min at 37 °C followed by 20 min at °80 C to inactivate the Bst enzyme. To determine the proper number of PCR cycles required to reach sufficient DNA concentration for sequencing but without overamplifying, 1 µL of library was amplified with qPCR and SYBR Green detection chemistry [primers as in Meyer and Kircher [3], conditions as in Allentoft, et al. [1]] and the cycle threshold (CT) values were recorded. The DNA library (21 µL) was then amplified and indexed in 50 µL PCR reactions using 25 µl of 2X KAPA HiFi HotStart Uracil + ReadyMix (KAPA Biosystems, Woburn, MA, USA) and 2 µl of 10 µM of each of Illumina's Multiplexing PCR primer in PE1.0 (use of different indexes among the three libraries). Thermocycling conditions were as follows: 45 s at 98 °C, followed by a sample-specific number of cycles (6–8 cycles) of 15 s at 98 °C, 30 s at 65 °C, and 30 s at 72 °C, and a final extension step of 30 s at 72 °C. The amplified library was purified with Agencourt AMPure XP magnetic beads (Beckman Coulter, Brea, CA, USA) with a beads:DNA ratio of 1.8:1, before being eluted in 30 µL EB buffer. Negative library controls constructed on H_2O were included, as well as libraries constructed on the negative extraction controls. Finally, the DNA concentration of each library was measured on an Agilent Bioanalyzer 2100 (Agilent, Santa Clara, CA, USA) and 'shotgun' sequenced on an Illumina NextSeq 500 platform using 75+6 bp single read chemistry (75 bp plus 6 bp index).

Supplementary Note S3. Details on ancient DNA data assessment

The sequences were de-multiplexed with a requirement of a full match of the six nucleotide indices that were used. Initial quality control of sequencing reads were performed using FastQC (http://www.bioinformatics.babraham.ac.uk/projects/fastqc/). The raw reads were trimmed for adapters and stretches of Ns using AdapterRemoval v.2.1.7; [4] and trimmed reads shorter than 30 bp were discarded. In order to test for the presence of genomic elephant DNA in the samples we mapped the reads against the reference genome of the African Savanna elephant (LoxAfr4; downloaded from ftp://ftp.broadinstitute.org/pub/assemblies/mammals/elephant/loxAfr4/), which is the only Elephantidae reference genome currently available. The trimmed reads were mapped using BWA v.0.7.15; [5] with default settings other than disabling the seed length (-l 1000) to allow for higher sensitivity [6]. Uniquely aligned reads were then identified by the XT, X1 tags, produced from the BWA alignment and filtered with the Linux command awk. SAMtools v.1.7; [7] was used to filter the uniquely mapped reads for mapping quality 25, sort them, and remove duplicate (PCR clonal) sequences. Local indel re-alignment was performed using the RealignerTargetCreator and IndelRealigner tools of GATK v.3.8-0; [8]. Read depth and coverage were determined using Qualimap 2 v.2.2.1; [9]. To investigate the level of DNA degradation in our samples, the mapped sequences were analyzed with mapDamage2.0 v.2.0.8; [10].

Supplementary Note S4. Details on Molecular species identification.

FastQ Screen v.0.13.0; [11] allows for the same sequencing library to be easily aligned to multiple reference genomes using Bowtie v.1.2.2; [12]. The percentage of raw reads that aligned a) uniquely to a single genome, b) to multiple places in the same genome, c) uniquely in multiple genomes, and d) to multiple places in multiple genomes can then be assessed. This method has been used successfully used for the molecular species identification of ancient specimens, including medieval parchments made of livestock-skin [13]. Since, a reference genome is currently available for only one elephant species, we used the reference mitochondrial genomes instead, which is available for all seven extinct and extant elephants. As input we used the adaptor-trimmed and length-filtered sequences after having the duplicated (clonal) sequences removed using SeqKit v.0.11.1; [14]. The filtered reads were competitively aligned to eight mitogenomes, including the African Savanna elephant (*L. africana*; refseq code NC_000934), the African forest elephant (*L. cyclotis*; NC_020759), the Asian elephant (*E. maximus*; NC_005129.2), the straight-tusked elephant (*P. antiquus*; NC_035230), the wooly mammoth (*Mammuthus primigenius*; NC_007596.2), the Columbian mammoth (*M. columbi*; NC_015529), the American mastodon (*Mammut americanum*; NC_035800), and the human rCRS (*Homo sapiens sapiens*; NC_012920). FastQ Screen alignment settings were modified to use Bowtie's 'end-to-end' algorithm and to allow zero mismatches between the read and the reference

genome. The mitochondrial DNA species assignment was also assessed via a metagenomics approach using MALT v.0.4.0; [15]. We used malt-build to construct a MALT index on the entire mitochondrial DNA database (mito_nt) of GenBank [16] and we align the (trimmed, filtered and de-duplicated) reads to the reference database using malt-run in BlastN mode and semi-global alignment with a minimum per cent identity (--minPercentIdentity) of 95, as well as a minimum support parameter (--minSupport) and a top per cent value (--topPercent) of 1. The generated RMA formatted output was used for interactive analysis of taxonomic composition in MEGAN v.6.10.11; [17].

Given that the two African elephant species are known to hybridize [18 and references therein], the mtDNA is not an adequate molecular marker to distinguish these two species as it is inherited only by females to their sprouts. In an attempt to distinguish between the two African elephant species, we repeated the FastQ Screen analysis using low coverage genomes of *L. cyclotis* and *L. loxodonta* that were mapped against LoxAfr4. The genome alignments have been generated by Palkopoulou, et al. [19] and were downloaded (in bam format; ERR2260495 and ERR2260496) from ENA (https://www.ebi.ac.uk/ena/data/view/PRJEB24361). The majority-rule consensus sequences were produced using ANGSD v.0.925-15-g334e8da; [20] with the minimum mapping and base quality thresholds set both to 30 and the minimum site depth set to 5.

Supplementary Note S5. Details on the F3-statistics analysis.

F3 statistics are defined as the product of allele frequency differences between population C to A and B, respectively: $F3(A, B; C) = \langle (c - a)(c - b) \rangle$, where $\langle \cdot \rangle$ denotes the average over all sites, and a, b and c denote the allele frequency for a given site in the three populations A, B, and C, respectively. If the F3 statistic is significantly negative (indicated by Z score < -3; p < 0.0001) there is evidence that the test population is admixed between the two source populations. The F3 statistic was calculated using the "threepop" v.0.1 subcommand of TreeMix v.1.13; [21]. Firstly, using the data from the tooth before the mapping we trimmed 2 bases at each sequence end using seqtk (https://github.com/lh3/seqtk), in order to reduce the bias inserted in our data due to cytosine deamination and then we repeated the mapping procedure as described above. Next, using bcftools v.1.9; [22] we generated a VCF file containing both variable and invariable sites (subcommands "mpileup" and "call") using LoxAfr4 as reference genome, with the minimum mapping and base quality thresholds set both to 30 and with adjust-MQ=50, as well as for normalization, site depth filtering (set to 3) and chromosome filtering (kept only the autosomal chromosomes 1-27) (subcommands "norm" and "filter"). Then, using the chromosome positions called for the tooth sample we repeat the above variant calling and filtering procedure for the two low-coverage

genome alignments. Using VCFtools v.0.1.16; [22] we merged all three VCF files, removed the indels (--remove-indels) and kept only the bi-allelic alleles (--min-alleles 2 --max-alleles 2). Finally, the merged and filtered VCF file was converted to TreeMix input file using the "populations" subcommand of Stacks 2 v.2.41; [23]. The input file contained 170 genome-wide SNPs that were present to all three individuals.

Supplementary Note S6. Details on mtDNA consensus sequence generation

A majority-rule consensus mitochondrial DNA sequence was produced using ANGSD v.0.925-15-g334e8da; [20] with the minimum mapping and base quality thresholds set both to 30. Using the same settings, we produced an IUPAC consensus mtDNA sequence, as well as another majority-rule consensus mtDNA sequence, but with also employing a minimum site depth filter of 3. By manually comparing these three sequences together with the reference mitogenome sequence we produced a manually corrected consensus sequence by implementing the following rules:

a) We kept all the sites with a depth of 3 or more, even if they differ compared to the reference sequence (they were considered as genuine variable sites; 97 cases in total).

b) For the sites with a depth of 2 or less and no IUPAC ambiguity letter observed, we masked the site (replaced with N; 42 cases in total) at the C/T and G/A SNPs (transitions).

c) For the sites with a depth of 2 and an IUPAC ambiguity letter (Y and R) observed at the C/T and G/A SNPs, respectively, we considered it as evidence of deamination damage and we changed the letter to C and G, respectively (26 cases in total).

In this way the final consensus mtDNA sequence is on the one hand less strict than the one with the site depth filter of 3 (e.g. monomorphic sites with site depth of 2 are not excluded) and on the other hand less error-prone than the ones without a site depth filter due to post-mortem C to T and G to A misincorporations caused by the degraded nature of ancient DNA. However, acknowledging that a reference bias may be inserted (through the third rule) and in order to evaluate the influence of this potential bias in the mtDNA phylogenetic placement of the sample, we performed the downstream phylogenetic analyses using both the manually corrected mtDNA consensus sequence and the one filtered with a site depth of 3.

Supplementary Note S7. Details on the alignment and mitochondrial DNA phylogenetic tree reconstruction.

The alignment among all the sequences in each dataset was performed with the MAFFT online service v.7; [24] with default values (alignment strategy selection was set to auto). An optimal set of partitions and substitution models was selected from all possible combinations of genes and tRNAs, considering all

substitution models available, under the Bayesian Information Criterion (BIC) in PartitionFinder 2 v.2.1.1; [25]. This analysis used the greedy search algorithm and linked branch lengths. In the phylogenetic reconstruction the mastodon and the Asian elephant were used as outgroup, in the complete and the partial mtDNA dataset, respectively. The alignment edges were trimmed in order to minimize the amount of missing data among individuals. The phylogenetic trees were reconstructed using a) the Neighbor Joining method [26] implemented in MEGA X (Kumar et al., 2018), with 1,000 bootstrap pseudo-replicates [27] to calculate the statistical significance of the resulted topology and b) the Maximum Likelihood method (RAxML-NG) implemented in the online tool RAxML BlackBox [28]. To ensure that the inferred ML tree was not a local optimum, 200 ML searches for each dataset were conducted (using 100 random and 100 parsimony starting trees) under the models of nucleotide substitution selected by PartitionFinder 2 for each subset (**Supplementary Tables 3-4**) and with linked branch lengths. Other than those mentioned, the rest of the parameters were set to default values. Bootstrapping was used to calculate the statistical significance with automatic bootstopping enabled (default cutoff value of 0.03).

Supplementary Table S1. List of elephant DNA sequences downloaded from GenBank and used in the present study. Dataset 1, full mitochondrion; Dataset 2, partial mitochondrion (continuous 4,258 bp fragment); Ref, reference mitochondrial genome.

Genbank Accession Code	Species	Dataset	Study
EF588275	*Elephas maximus*	Dataset 1	[29]
AB443879	*Loxodonta africana*	Dataset 1	[30]
DQ316069	*Loxodonta africana*	Dataset 1	[31]
KY616974	*Loxodonta africana*	Dataset 1	[32]
KY616977	*Loxodonta africana*	Dataset 1	[32]
KY616982	*Loxodonta africana*	Dataset 1	[32]
NC_000934 (Ref)	*Loxodonta africana*	Dataset 1	[33]
JN673263	*Loxodonta cyclotis*	Dataset 1	[34]
KJ557423	*Loxodonta cyclotis*	Dataset 1	[35]
KJ557424	*Loxodonta cyclotis*	Dataset 1	[35]
KY616975	*Loxodonta cyclotis*	Dataset 1	[32]
KY616976	*Loxodonta cyclotis*	Dataset 1	[32]
KY616978	*Loxodonta cyclotis*	Dataset 1	[32]
KY616979	*Loxodonta cyclotis*	Dataset 1	[32]
KY616980	*Loxodonta cyclotis*	Dataset 1	[32]
KY616981	*Loxodonta cyclotis*	Dataset 1	[32]
NC_020759 (Ref)	*Loxodonta cyclotis*	Dataset 1	[34]
EF632344	*Mammut americanum*	Dataset 1	[36]
NC_035800 (Ref)	*Mammut americanum*	Dataset 1	[37]
NC_015529 (Ref)	*Mammuthus columbi*	Dataset 1	[38]
AP008987	*Mammuthus primigenius*	Dataset 1	Unpublished (Direct submission to GenBank)
EU153444	*Mammuthus primigenius*	Dataset 1	[39]
NC_007596 (Ref)	*Mammuthus primigenius*	Dataset 1	[40]
KY499556	*Palaeoloxodon antiquus*	Dataset 1	[32]
KY499557	*Palaeoloxodon antiquus*	Dataset 1	[32]
KY499558	*Palaeoloxodon antiquus*	Dataset 1	[32]
NC_035230 (Ref)	*Palaeoloxodon antiquus*	Dataset 1	[32]
NC_005129 (Ref)	*Elephas maximus*	Dataset 1, Dataset 2 (trimmed)	[31]
JQ438119 - JQ438259	*Loxodonta africana*	Dataset 2	[41]
JQ438334 - JQ438500	*Loxodonta africana*	Dataset 2	[41]
JQ438519 - JQ438771	*Loxodonta africana*	Dataset 2	[41]
JQ438260 - JQ438313	*Loxodonta cyclotis*	Dataset 2	[41]
JQ438501 - JQ438518	*Loxodonta cyclotis*	Dataset 2	[41]
JQ438314 - JQ438333	*Loxodonta cyclotis* x *Loxodonta africana* (hybrid)	Dataset 2	[41]

Supplementary Table S2. Sequencing and mapping to reference genome statistics of the three extracts from the dental elephant root.

Sample	Total	Retained	Mapped	Non-clonal	Endogenous (%)	Efficiency (%)
ADNA_100098_1	1,666,083	1,601,801	1,016,835	1,000,707	63.48	60.06
ADNA_100098_2	858,409	813,719	374,476	368,511	46.02	42.92
ADNA_100098_3	581,418	544,352	128,689	126,520	23.64	21.76
ADNA_100098 (combined)	3,105,910	2,959,872	1,519,993	1,494,954	51.35	48.13

Total, total generated sequences; Retained, number of sequences retained after adapter residues and consecutive Ns trimming and removing <30bp sequences after trimming; Mapped, number of sequences uniquely aligned to the *L. africana* reference genome (LoxAfr4); Non-clonal, number of sequences retained after removing identical sequences (clones) within each library; Endogenous, the percentage of uniquely mapped reads among retained number; Efficiency, the percentage of non-clonal uniquely aligned sequences among total number of generated sequences.

Supplementary Table S3. Molecular preservation of the three extracts from the dental elephant root.

Sample	C-T (%)	Average Length (bp)
ADNA_100098_1	7.69	57.81
ADNA_100098_2	9.51	60.31
ADNA_100098_3	11.00	58.31
ADNA_100098 (combined)	8.40	58.47

C-T, percentage of sequences showing DNA deamination damage at position 1 (5'- end); Av. Length, average length (bp) of the uniquely aligned sequences.

Supplementary Table S4. Alignment descriptive statistics.

Dataset	Alignment length (bp)	Variable sites	Parsimony informative sites
Full MT	16,025	2,863 (17.86%)	2,694 (16.81%)
Partial MT	4,258	526 (12.35%)	374 (8.78%)

Supplementary Table S5. The subsets selected by PartitionFinder 2 for the full mitochondrial dataset, with information on the best nucleotide substitution model fitted to describe the data of each subset, the number of sites of each subset, and the user pre-defined partitions merged under the same subset.

Subset	Best Model	Number of sites	Partition names
1	TRN+I+G	2425	ATP8_pos2, ND6_pos2, ND3_pos2, ATP6_pos1, tRNA-Ser2, tRNA-Phee, ND6_pos1, ND5_pos3, ND2_pos1, tRNA-Thr, tRNA-Glu, ND4_pos2
2	TRN+I+G	3844	tRNA-Asn, tRNA-Lys, tRNA-Gly, ATP8_pos3, tRNA-Trp, tRNA-Ala, 12S-rRNA, tRNA-Ile, tRNA-Leu1, tRNA-Val, tRNA-Gln, tRNA-His, tRNA-Arg, 16S-rRNA, tRNA-Tyr, tRNA-Met, tRNA-Cys, tRNA-Ser1, tRNA-Pro, tRNA-Leu2, tRNA-Asp
3	K80+I	1842	COX3_pos1, COX1_pos2, ND4L_pos3, cytb_pos1, rep, COX2_pos2, ND1_pos1
4	HKY+I	3533	COX1_pos3, COX2_pos3, cytb_pos2, COX3_pos2, ND3_pos3, ND5_pos1, ND4_pos3, ND2_pos2, ATP6_pos2, ND1_pos2, ND4L_pos1
5	TRN+G	2396	ND2_pos3, ND6_pos3, ND1_pos3, ND5_pos2, cytb_pos3, ND4_pos1, ND3_pos1
6	TRN	1380	COX1_pos1, COX2_pos1, ATP8_pos1, ATP6_pos3, ND4L_pos2, COX3_pos3
7	HKY+I+G	605	D-loop

Supplementary Table S6. The subsets selected by PartitionFinder 2 for the partial mitochondrial dataset (continuous 4,258 bp fragment), with information on the best nucleotide substitution model fitted to describe the data of each subset, the number of sites of each subset, and the user pre-defined partitions merged under the same subset.

Subset	Best Model	Number of sites	Partition names
1	TRN+G	1328	ND6_pos3, ND6_pos1, cytb_pos3, ND5_pos1
2	HKY+I	1364	tRNA-Pro, cytb_pos1, tRNA-Thr, ND6_pos2, ND5_pos2, tRNA-Glu
3	HKY+I	976	ND5_pos3, cytb_pos2
4	HKY+I+G	590	D-loop

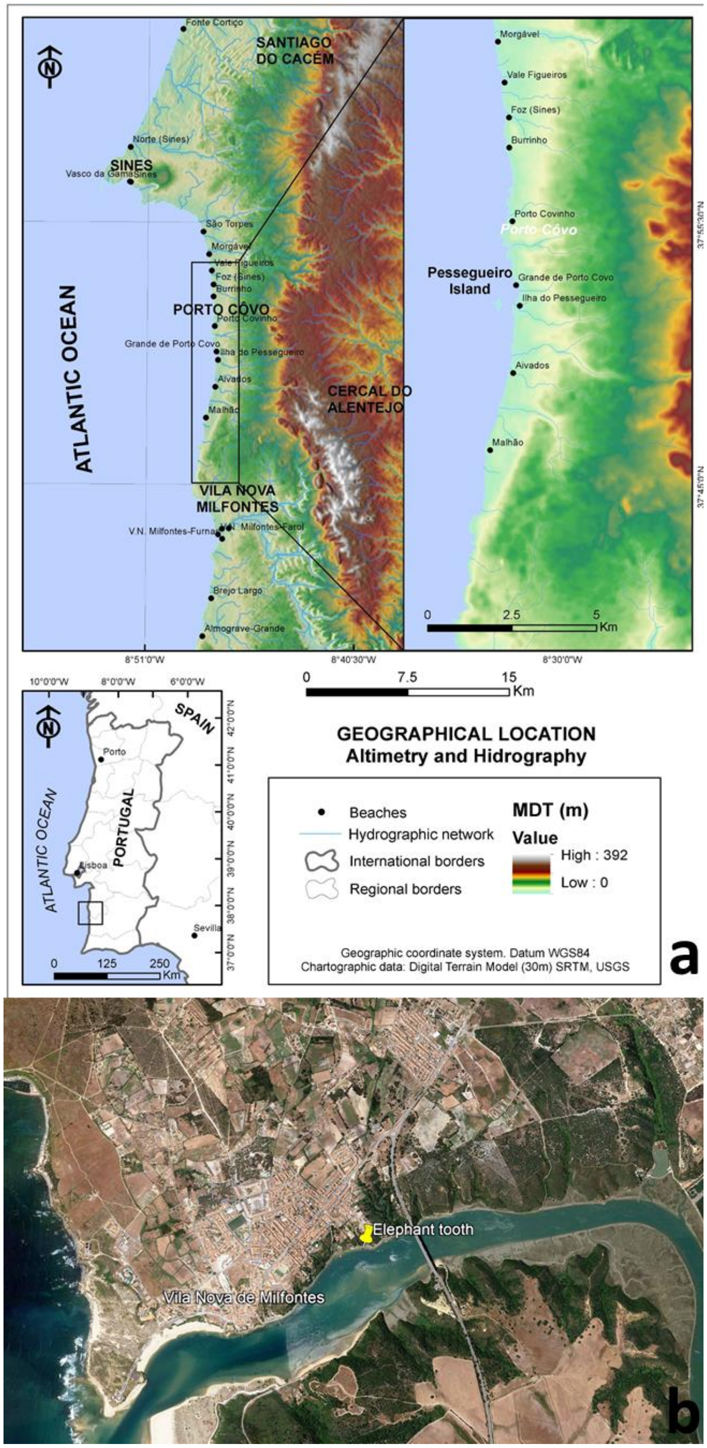

Supplementary Figure 1. Geographical location of the molar tooth, in the southwestern coast of Portugal, near the town of Vila Nova de Milfontes (a), with the location of coastal sites with upper Pleistocene *Palaeoloxodon antiquus* remains (box) after [42]. The map was made in ArcGIS 9 using a Digital Terrain Model (30 m) provided by USGS for free as shapefile in https://www.usgs.gov/centers/eros/science/usgs-eros-archive-digital-elevation-shuttle-radar-topography-mission-srtm-non?qt-science_center_objects=0#qt-science_center_objects. Satellite image (made in Google Earth 7.3.3.7786) of the exact location of the finding in the mouth of the Mira River (b).

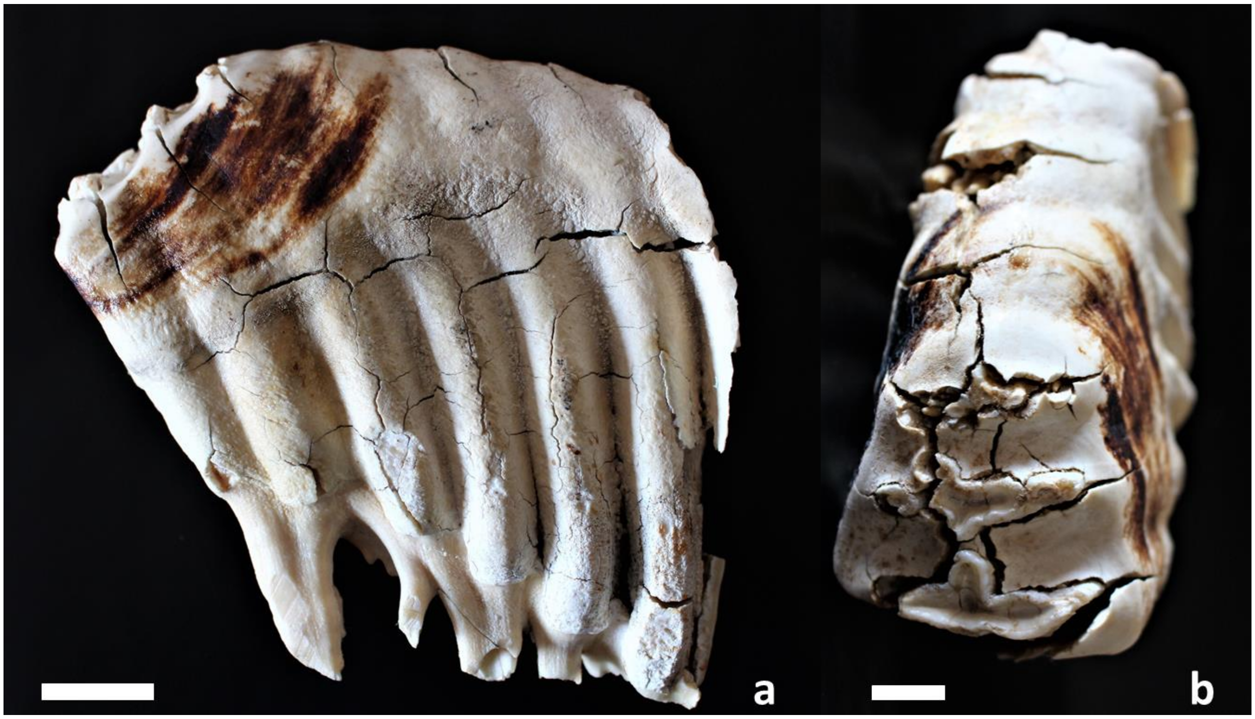

Supplementary Figure 2. Labial (a) and occlusal (b) views of the M3 identified as *Loxodonta cyclotis*; scale bar = 20 mm. The photo was taken by Silvério Figueiredo.

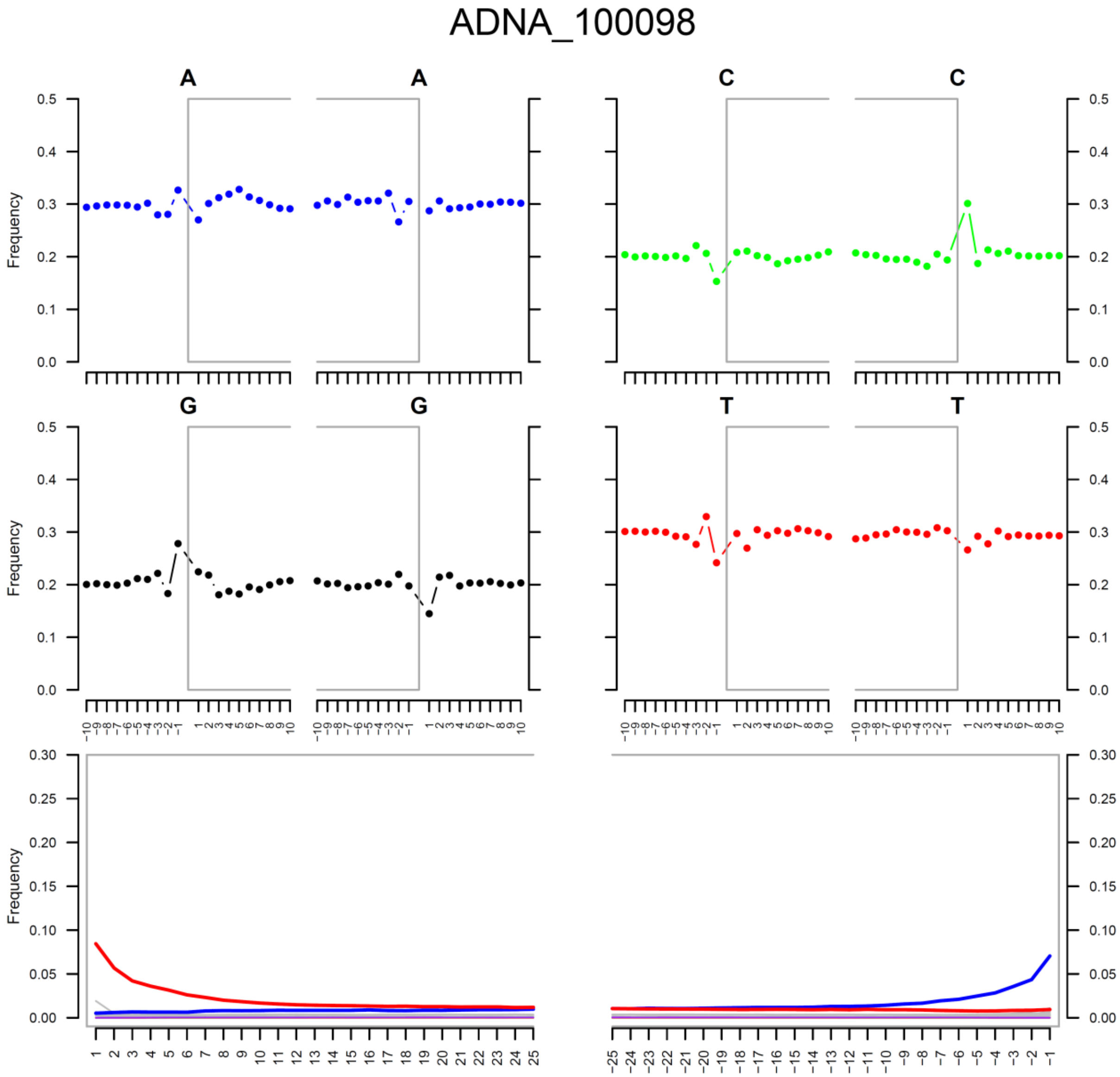

Supplementary Figure 3. DNA damage based on shotgun sequencing data of the combined data generated from the elephant tooth, using the *L. africana* (LoxAfr4) reference genome for the mapping. The four upper mini-plots show the base frequency outside, and in the sequencing read (the open grey box corresponds to the read). The bottom plots are the positions' specific substitutions from the 5' (left) and the 3' end (right). The following color codes are used in the bottom plots: Red, C to T substitutions; Blue, G to A substitutions; Grey, all other substitutions; Orange, soft-clipped bases; Green, deletions relative to the reference; Purple, insertions relative to the reference.

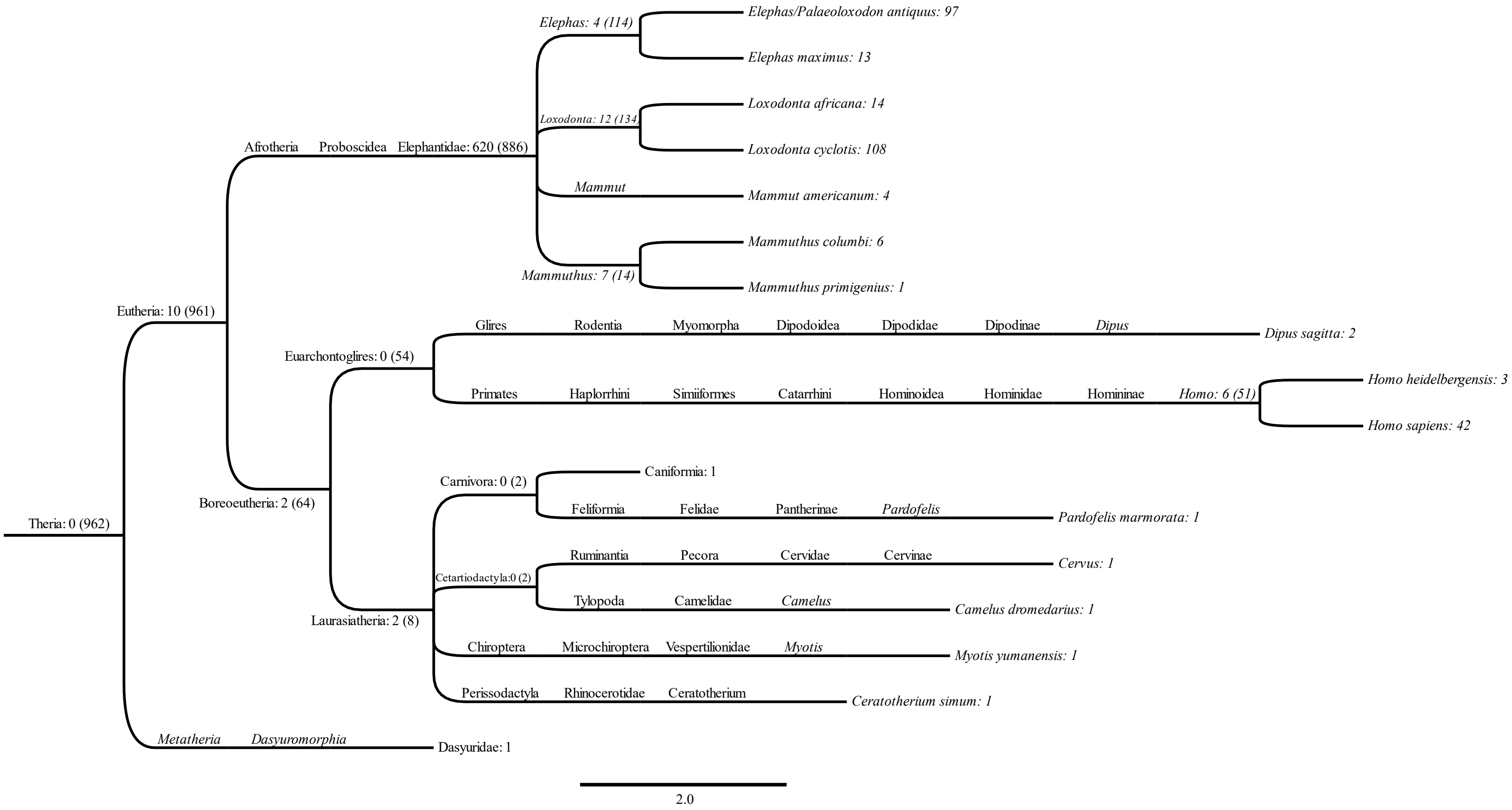

Supplementary Figure 4. Metagenomic (MALT) assignment of the generated sequences from the elephant tooth. The reference database included the entire mitochondrial database of Genbank (mito_nt). The taxonomic tree and names were provided by NCBI. The numbers next to taxa names correspond to the number of sequences assigned to the given taxon (tree node or tip) *per se*, whereas the numbers in brackets correspond to the summarized number of sequences assigned to all taxa downstream of the given taxon (tree node).

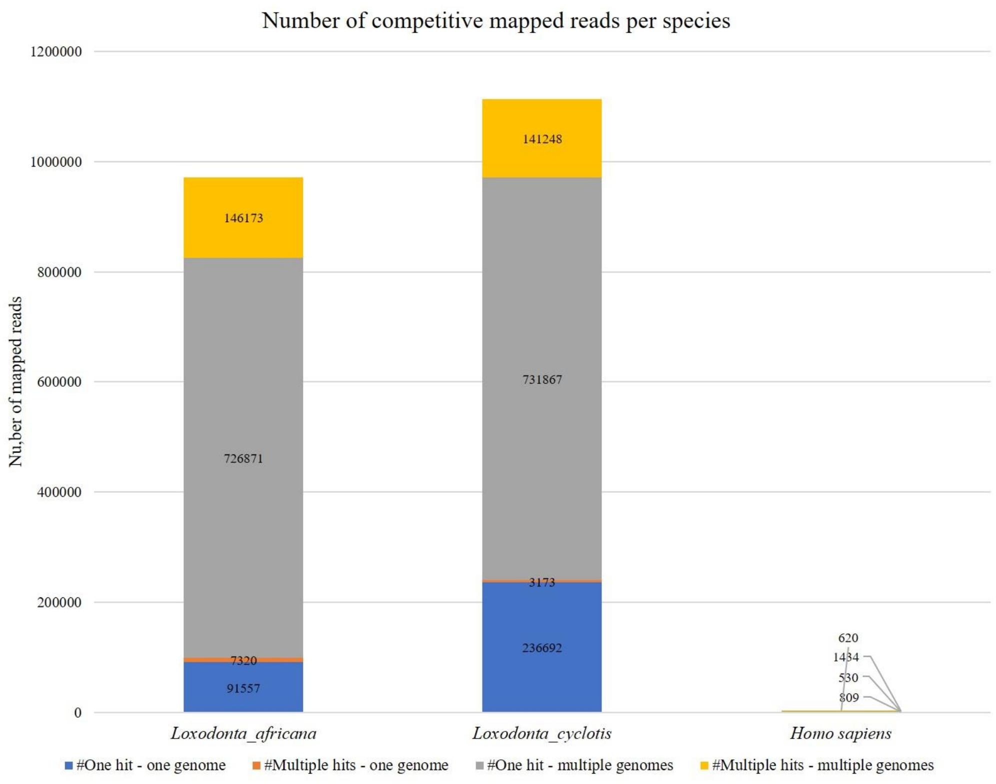

Supplementary Figure 5. Competitive mapping (FastQ Screen analysis) of the generated sequences from the tooth against two low-coverage African elephant genomes, as well as against the reference genome of humans.

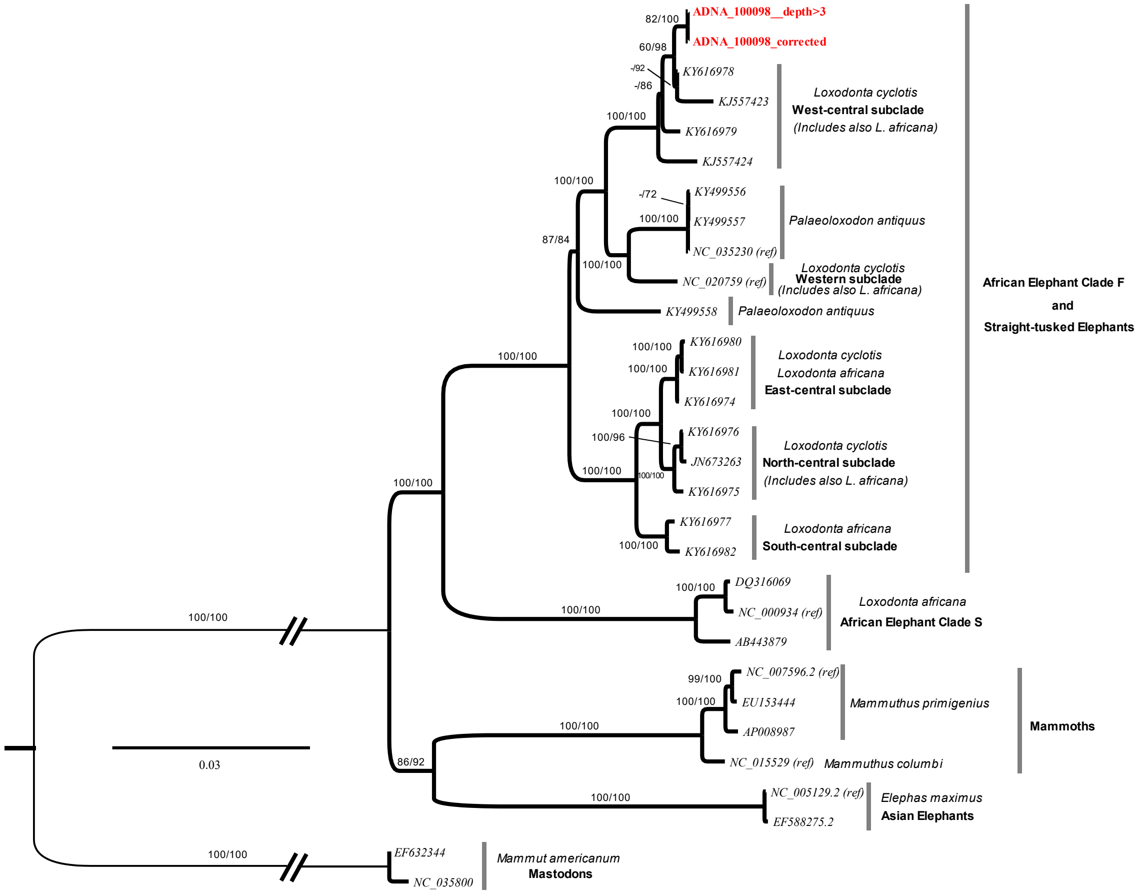

Supplementary Figure 6. The placement of the tooth (ADNA_100098) mtDNA consensus sequences in the Maximum Likelihood phylogenetic tree of Elephantidae using the whole mtDNA genome dataset. The numbers on the branches correspond to the bootstrap support (NJ/ML). The reference sequence of each species is noted (ref). The clade/subclade nomenclature and the comment inside brackets is based on Ishida, et al. [41] declaring the grouping of conplastic (within individual mitogenomic incongruence; see discussion) *L. africana* individuals with pure *L. cyclotis* ones.

Supplementary Information References

1 Allentoft, M. E. *et al.* Population genomics of Bronze Age Eurasia. *Nature* **522**, 167, doi:10.1038/nature14507 (2015).
2 Damgaard, P. B. *et al.* Improving access to endogenous DNA in ancient bones and teeth. *Sci Rep* **5**, 11184, doi:10.1038/srep11184 (2015).
3 Meyer, M. & Kircher, M. Illumina Sequencing Library Preparation for Highly Multiplexed Target Capture and Sequencing. *Cold Spring Harbor Protocols* **2010**, pdb.prot5448, doi:10.1101/pdb.prot5448 (2010).
4 Lindgreen, S. AdapterRemoval: easy cleaning of next-generation sequencing reads. *BMC Res Notes* **5**, 337-337, doi:10.1186/1756-0500-5-337 (2012).
5 Li, H. & Durbin, R. Fast and accurate short read alignment with Burrows–Wheeler transform. *Bioinformatics* **25**, 1754-1760, doi:10.1093/bioinformatics/btp324 (2009).
6 Schubert, M. *et al.* Improving ancient DNA read mapping against modern reference genomes. *BMC Genomics* **13**, 178-178, doi:10.1186/1471-2164-13-178 (2012).
7 Li, H. *et al.* The Sequence Alignment/Map format and SAMtools. *Bioinformatics* **25**, 2078-2079, doi:10.1093/bioinformatics/btp352 (2009).
8 McKenna, A. *et al.* The Genome Analysis Toolkit: A MapReduce framework for analyzing next-generation DNA sequencing data. *Genome Res.* **20**, 1297-1303, doi:10.1101/gr.107524.110 (2010).
9 Okonechnikov, K., Conesa, A. & García-Alcalde, F. Qualimap 2: advanced multi-sample quality control for high-throughput sequencing data. *Bioinformatics* **32**, 292-294, doi:10.1093/bioinformatics/btv566 (2016).
10 Jónsson, H., Ginolhac, A., Schubert, M., Johnson, P. L. F. & Orlando, L. mapDamage2.0: fast approximate Bayesian estimates of ancient DNA damage parameters. *Bioinformatics* **29**, 1682-1684, doi:10.1093/bioinformatics/btt193 (2013).
11 Wingett, S. W. & Andrews, S. FastQ Screen: A tool for multi-genome mapping and quality control. *F1000Res.* **7**, 1338-1338, doi:10.12688/f1000research.15931.2 (2018).
12 Langmead, B., Trapnell, C., Pop, M. & Salzberg, S. L. Ultrafast and memory-efficient alignment of short DNA sequences to the human genome. *Genome Biol.* **10**, R25, doi:10.1186/gb-2009-10-3-r25 (2009).
13 Teasdale, M. D. *et al.* Paging through history: parchment as a reservoir of ancient DNA for next generation sequencing. *Philos. Trans. R. Soc. Lond. B. Biol. Sci.* **370**, 20130379-20130379, doi:10.1098/rstb.2013.0379 (2015).
14 Shen, W., Le, S., Li, Y. & Hu, F. SeqKit: A Cross-Platform and Ultrafast Toolkit for FASTA/Q File Manipulation. *PLoS ONE* **11**, e0163962, doi:10.1371/journal.pone.0163962 (2016).
15 Herbig, A. *et al.* MALT: Fast alignment and analysis of metagenomic DNA sequence data applied to the Tyrolean Iceman. *bioRxiv*, 050559, doi:10.1101/050559 (2016).
16 Benson, D. A. *et al.* GenBank. *Nucleic Acids Res.* **46**, D41-d47, doi:10.1093/nar/gkx1094 (2018).
17 Huson, D. H., Auch, A. F., Qi, J. & Schuster, S. C. MEGAN analysis of metagenomic data. *Genome res.* **17**, 377-386, doi:10.1101/gr.5969107 (2007).
18 Mondol, S. *et al.* New evidence for hybrid zones of forest and savanna elephants in Central and West Africa. *Mol. Ecol.* **24**, 6134-6147, doi:10.1111/mec.13472 (2015).
19 Palkopoulou, E. *et al.* A comprehensive genomic history of extinct and living elephants. *PNAS* **115**, E2566-e2574, doi:10.1073/pnas.1720554115 (2018).
20 Korneliussen, T. S., Albrechtsen, A. & Nielsen, R. ANGSD: Analysis of Next Generation Sequencing Data. *BMC bioinformatics* **15**, 356, doi:10.1186/s12859-014-0356-4 (2014).
21 Pickrell, J. K. & Pritchard, J. K. Inference of Population Splits and Mixtures from Genome-Wide Allele Frequency Data. *PLoS Genetics* **8**, e1002967, doi:10.1371/journal.pgen.1002967 (2012).

22 Danecek, P. *et al.* The variant call format and VCFtools. *Bioinformatics* **27**, 2156-2158, doi:10.1093/bioinformatics/btr330 (2011).
23 Rochette, N. C., Rivera-Colón, A. G. & Catchen, J. M. Stacks 2: Analytical methods for paired-end sequencing improve RADseq-based population genomics. *Mol. Ecol.* **28**, 4737-4754, doi:10.1111/mec.15253 (2019).
24 Katoh, K., Rozewicki, J. & Yamada, K. D. MAFFT online service: multiple sequence alignment, interactive sequence choice and visualization. *Brief. Bioinformatics* **20**, 1160-1166, doi:10.1093/bib/bbx108 (2019).
25 Lanfear, R., Frandsen, P. B., Wright, A. M., Senfeld, T. & Calcott, B. PartitionFinder 2: New Methods for Selecting Partitioned Models of Evolution for Molecular and Morphological Phylogenetic Analyses. *Mol. Biol. Evol.* **34**, 772-773, doi:10.1093/molbev/msw260 (2017).
26 Saitou, N. & Nei, M. The neighbor-joining method: a new method for reconstructing phylogenetic trees. *Mol. Biol. Evol.* **4**, 406-425, doi:10.1093/oxfordjournals.molbev.a040454 (1987).
27 Felsenstein, J. Confidence limits on phylogenies: An approach using the bootstrap. *Evolution; international journal of organic evolution* **39**, 783-791, doi:10.1111/j.1558-5646.1985.tb00420.x (1985).
28 Kozlov, A. M., Darriba, D., Flouri, T., Morel, B. & Stamatakis, A. RAxML-NG: a fast, scalable and user-friendly tool for maximum likelihood phylogenetic inference. *Bioinformatics* **35**, 4453-4455, doi:10.1093/bioinformatics/btz305 (2019).
29 Maikaew, U., Pinyopummin, A., Wajjwalku, W., Siripholvat, V. & Mahasawankul, S. The Study of Nucleotides Arrangement of Mitochondrial DNA in Thai Elephants (*Elephas maximus indicus*). *KKU Vet.* **17**, 11-21 (2011).
30 Murata, Y. *et al.* Chronology of the extant African elephant species and case study of the species identification of the small African elephant with the molecular phylogenetic method. *Gene* **441**, 176-186, doi:10.1016/j.gene.2009.01.014 (2009).
31 Rogaev, E. I. *et al.* Complete mitochondrial genome and phylogeny of Pleistocene mammoth *Mammuthus primigenius*. *PLoS Biol.* **4**, e73, doi:10.1371/journal.pbio.0040073 (2006).
32 Meyer, M. *et al.* Palaeogenomes of Eurasian straight-tusked elephants challenge the current view of elephant evolution. *eLife* **6**, e25413, doi:10.7554/eLife.25413 (2017).
33 Hauf, J., Waddell, P. J., Chalwatzis, N., Joger, U. & Zimmermann, F. K. The complete mitochondrial genome sequence of the African elephant (*Loxodonta africana*), phylogenetic relationships of Proboscidea to other mammals and D-loop heteroplasmy. *Zoology (Jena)* **102**, 184-195 (2000).
34 Brandt, A. L., Ishida, Y., Georgiadis, N. J. & Roca, A. L. Forest elephant mitochondrial genomes reveal that elephantid diversification in Africa tracked climate transitions. *Mol. Ecol.* **21**, 1175-1189, doi:10.1111/j.1365-294X.2012.05461.x (2012).
35 Finch, T. M., Zhao, N., Korkin, D., Frederick, K. H. & Eggert, L. S. Evidence of positive selection in mitochondrial complexes I and V of the African elephant. *PLoS One* **9**, e92587, doi:10.1371/journal.pone.0092587 (2014).
36 Rohland, N. *et al.* Proboscidean mitogenomics: chronology and mode of elephant evolution using mastodon as outgroup. *PLoS Biol.* **5**, e207, doi:10.1371/journal.pbio.0050207 (2007).
37 Claesson, S., Baleka, S., Hofreiter, M. & Widga, C. The contribution of Late Pleistocene megafauna finds to submerged archaeology and the interpretation of ancient coastal landscapes. *J. Archaeol. Sci. Rep.* **15**, 290-298, doi:https://doi.org/10.1016/j.jasrep.2017.08.007 (2017).
38 Enk, J. *et al.* Complete Columbian mammoth mitogenome suggests interbreeding with woolly mammoths. *Genome Biol.* **12**, R51, doi:10.1186/gb-2011-12-5-r51 (2011).
39 Gilbert, M. T. *et al.* Whole-genome shotgun sequencing of mitochondria from ancient hair shafts. *Science* **317**, 1927-1930, doi:10.1126/science.1146971 (2007).
40 Krause, J. *et al.* Multiplex amplification of the mammoth mitochondrial genome and the evolution of Elephantidae. *Nature* **439**, 724-727, doi:10.1038/nature04432 (2006).

41 Ishida, Y., Georgiadis, N. J., Hondo, T. & Roca, A. L. Triangulating the provenance of African elephants using mitochondrial DNA. *Evol. Appl.* **6**, 253-265, doi:10.1111/j.1752-4571.2012.00286.x (2013).
42 Neto de Carvalho, C., Figueiredo, S. & Belo, J. Vertebrate tracks and trackways from the pleistocene eolianites of SW Portugal. *Comun. Geol.* **103**, 101-116 (2016).
